# Research disruption during PhD studies and its impact on mental health: Implications for research and university policy

**DOI:** 10.1371/journal.pone.0291555

**Published:** 2023-10-18

**Authors:** Maria Aristeidou, Angela Aristidou

**Affiliations:** 1 Institute for Educational Technology, The Open University, Milton Keynes, Buckinghamshire, United Kingdom; 2 UCL School of Management, London, United Kingdom; Mettu University, ETHIOPIA

## Abstract

Research policy observers are increasingly concerned about the impact of the disruption caused by the Covid-19 pandemic on university research. Yet we know little about the effect of this disruption, specifically on PhD students, their mental health, and their research progress. This study drew from survey responses of UK PhD students during the Covid-19 pandemic. We explored evidence of depression and coping behaviour *(N = 1780)*, and assessed factors relating to demographics, PhD characteristics, Covid-19-associated personal circumstances, and significant life events that could explain PhD student depression during the research disruption *(N = 1433)*. The majority of the study population (86%) reported a negative effect on their research progress during the pandemic. Results based on eight mental health symptoms (PHQ-8) showed that three in four PhD students experienced significant depression. Live-in children and lack of funding were among the most significant factors associated with developing depression. Engaging in approach coping behaviours (i.e., those alleviating the problem directly) related to lower levels of depression. By assessing the impact of research disruption on the UK PhD researcher community, our findings indicate policies to manage short-term risks but also build resilience in academic communities against current and future disruptions.

## Introduction

The abrupt outbreak in January 2020 and the global proliferation of a novel virus (Covid-19) has created a crisis for many sectors, including the international higher education (HE) sector [1] that continues during the ‘post-pandemic’ period. A point of particular alarm for HE leaders, policy observers, and governments is the disruption to the typical flow and pace of university research activity. While research related to Covid-19 is still in overdrive, other research was slowed or stopped due to worldwide physical distancing measures to contain the virus’ spread (e.g., sudden campus and laboratory closures, mobility restrictions, stay-at-home orders) [2]. The resulting ‘drop in research work’ is suggested to have a detrimental impact on the HE sector on the ‘research and innovation pipeline’ [3], and on ‘research capacity, innovation and research impact’ [4].

As research and university policies internationally are being (re)shaped at a rapid pace in efforts to meet the challenge of university research disruption [5], we contribute to academic and policy conversations by examining the effect of the research disruption on the mental health of PhD students. A considerable body of research acknowledges the role of PhD students in the innovation process, in knowledge creation and diffusion (e.g., [6]) and further posits that the period of one’s PhD program is key to early career success and research productivity (e.g., [7]). These outcomes, which matter to research policy, have been linked to PhD student mental health [8–10]. In those times of relative stability, research had additionally demonstrated the higher prevalence of mental health issues amongst the PhD student population across research disciplines, as compared to other students within academia [9] and the general population [9, 11, 12]. In the period since Covid-19 disrupted our social and economic lives, depression levels in the general population have been exacerbated globally [13, 14]. These trends suggested that the already high prevalence of poor mental health in PhD students is likely to be further exacerbated during the pandemic. Indeed, as reported in early studies on research students’ experience of the Covid-19 pandemic (e.g., [15]) and the post-pandemic period (e.g., [16]) the impact on students’ mental wellbeing has been significant, with students suggesting a number of support measures at institutional and national level.

Ignoring, at this critical moment, the increased likelihood of poor mental health in PhD students may jeopardize research capacity and HE competitiveness for years to come. Therefore, there is a pressing need to identify–within the PhD student population–those whose mental health is more affected by the research disruption, so that policies and assistance can be timelier and more targeted. Additionally, by understanding more clearly the factors that may contribute to poor mental health, and their interrelationships (presented in Methods), policymakers and HE leaders may be better placed to tackle, and ultimately overcome, this and future research disruptions.

Motivated by the current lack of an empirical basis for insights into PhD students’ mental health during the pandemic-induced disruption, we collected survey data contemporaneously during July 2020. Our 1780 survey respondents are PhD students in 94 UK Universities, across the natural and social sciences and across PhD stages. Our study has three objectives: first, to explore mental health prevalence (depression) and coping behaviour in a large-scale representative sample of PhD students in the UK (O1); second, to evaluate the relationships among mental health prevalence and coping behaviour (O2); third, to identify factors that increase the likelihood of poor PhD student mental health during the period of research disruption (O3). Our study extends previous research on mental health in the HE sector by considering the dynamics of severe disruption, as opposed to the dynamics of relative stability, on PhD students’ mental health, performance satisfaction, and coping behaviours.

## Background and literature review

### UK PhD students’ mental health in times of disruption

In the UK, there are approximately 100,000 postgraduate students completing doctoral research [17]. Since 2018, significant government funding has been targeted at developing insights into supporting UK PhD students’ mental health [18]. Still, with the exception of Byrom et al. [11], published research on PhD students’ mental health in the UK exhibits the same limitations as the international research: It reflects discipline- or institution-related specificity (e.g., [19]) or utilizes samples of early career researchers in general (e.g., [20]).

Early findings on postgraduate research students’ wellbeing during the pandemic showed that only a small proportion of them are in good mental health wellbeing (28%) while the rest demonstrate possible or probable depression or anxiety [15]. Goldstone and Zhang [15] further highlight the differences among student groups with, for example, students with disabilities or caring responsibilities or female students having lower levels of mental wellbeing. The post-pandemic findings have been more promising, as only about one in four students were at risk of experiencing mental health issues [16].

In response to the Covid-19 research disruption, substantive actions have been taken by the HE sector and the UK Government to disseminate approaches deployed by UK universities to support student mental health (e.g., [18]) and to update mental health frameworks for UK universities (e.g., [4]), but so far, mitigation activities have been targeting mental health for UK university students broadly, not UK PhD students specifically.

Overcoming the paucity of evidence on UK PhD students’ mental health during the pandemic is a crucial first step to drawing strong conclusions on the prevalence and determinants of mental health issues and ways to mitigate them specific to the PhD population. For example, policy recommendations by UK postgraduate respondents during the pandemic [15] focused mainly on financial support, such as extensions to their funded period of study and tuition and visa fee support (including waivers to fees). To develop an overarching framework specific to the Objectives of our study, we synthesize insights from the international literature on PhD student mental health conducted in the period before the research disruption.

### International research on PhD student mental health in times of relative stability

In the international literature examining mental health specifically for PhD students (see the systematic review in [21], the issue of mental health for PhD students is acknowledged to be multidimensional and complex [10]. In this growing research area, some address mental health as an aspect of the broader ‘health’ of the PhD students (e.g., [22]), some focus on psychological distress [23], while others take depression as a specific manifestation of distress [9, 24]. The latter is particularly interesting because depression within the PhD population in these studies is often assessed with standardised questionnaires (e.g., PHQ, see below) that allow for developing comparative insights. It is also the approach adopted by the only global survey of PhD students’ mental health by Evans et al. [12], showing that 39% of PhD students report moderate-to-severe depression, significantly more than the general population.

### Literature on PhD student’s mental health determinants in times of relative stability

Past literature on PhD students’ mental health offers insights into the determinants of PhD students’ mental health in times of stability, which may help understand the relationships we want to examine between PhD mental health, performance satisfaction and coping in times of research disruption.

First, past studies evidence the influence of PhD students’ personal lives on poor mental health. PhD students with children or with partners are less likely to have or develop psychological distress [9]. The normalcy of family roles is a much-needed antidote to the known pressures of a PhD program [25] and might even protect against mental health problems [22, 26]. Other aspects of PhD students’ personal lives, such as significant life events (e.g., severe problems in personal relationships or severe illness of the student or someone close to them), have been linked to dissatisfaction with their research progress [24]. Research progress is defined as students’ perception of their progress in the completion of their degree [27] and is linked to their mental health. Dissatisfaction is tied to negative outcomes, such as attrition and delay [28], but also to lower productivity and mental health problems, such as worry, anxiety, exhaustion, and stress [29]. Related to this, Levecque and colleagues [9] observed that PhD students expressing a high interest in an academic career are in better mental health than those with no or little interest in remaining in academia.

Second, gender was the key personal factor that emerged as a determinant for mental health in past studies: PhD students who self-identify as female report greater clinical [9, 30] and non-clinical problems with their mental health [23, 31]. This is explained through the additional pressure women report on their professional and personal lives [23].

Third, past studies argue that each PhD phase presents PhD students with specific sets of challenges and should thus be explored discreetly in relation to mental health [32]. Still, the evidence on the link between the PhD phase (or the year of study as a proxy for the PhD phase) and mental health is inconclusive. Barry et al.’s [33] survey reports no connection between the PhD phase and depression levels in an Australian PhD population. However, Levecque et al. [9] report high degrees of depression in the early PhD stage of students in Belgium, and a global survey of PhD students across countries and disciplines shows that depression likelihood increases as the PhD program progresses [32].

Fourth, past research offers strong evidence that financial concerns impact PhD students’ mental health negatively. In a study by El-Ghoroury et al. [34], 63.9% of PhD students cited debt or financial issues as a cause for poor wellbeing and cited financial constraints as the major barrier to improving their wellness (through social interactions, outside-PhD activities, etc). Even uncertainty about funding was shown to predict poor mental health [9]. To this end, Geven et al. [35] explored packages of reforms in a pre-pandemic graduate school programme, including an extension of the grant period, and indicated that such policies can increase students’ completion rates to up to 20%.

Finally, age is not shown to be associated with mental health [9], but numerous studies found that having children, particularly for female PhD students and in Science-Technology-Engineering-Maths (STEM) disciplines [36], consistently corresponds with heightened stress [37]. However, a specific examination of the relationship between children and mental health indicates that PhD students with one or more children in the household showed significantly lower odds of having or developing a common psychiatric disorder [9]. Further, parenting and, in particular, motherhood during doctorate studies contribute to the development of students’ coping mechanisms that allows them to succeed in a balance in both worlds [38].

### Past research insights into PhD mental health and coping

Past research explored how PhD students may “cope” with stressors and thus mitigate poor mental health [39]. Studies identify the importance of social interactions (e.g., [22]); balancing life demands (e.g., [16]), reaching out for social support (e.g., [40]) sometimes through peer relationships (e.g., [10, 39]); and ‘planning’ (e.g., [22]); As invaluable as these insights are, drawing comparisons between these findings is difficult because often the identification of coping styles or strategies was not the focus of these studies, making it difficult to draw fine-grained conclusions as to their effect on PhD students’ mental health.

There is, however, a long tradition of research on coping for physiological wellbeing that provides standardised measures for individuals’ coping and their link to mental health [41]. The most widely used measurement instrument in the literature reviewed is the COPE Inventory, which allows researchers to assess how people cope in a variety of stressful situations, including in HE for students [42–44], making it particularly relevant to the context and sample under investigation in our study of PhD students. Additionally, COPE allows for the identification of consistent ways of coping, which provides predictive validity across a range of situations. Predictive validity is desired when examining the role of coping in relation to mental health. Indeed, multiple studies have linked the COPE measurement to mental health outcomes (e.g., [45, 46]), including depression [43], which is a focus of our study.

## Data and methods

### Participants

For the current study, we recruited participants that were active PhD students from March to July 2020 at any stage of their research to take part in an online survey. The survey ran between the 31st of July and the 23rd of August 2020, with the aim of capturing the potential impact of the Covid-19 disruption during the first lockdown on their research progress and mental health. The use of online surveys to assess the scope of mental health problems is particularly appropriate during the Covid-19 outbreak [47]. The current study has been reviewed by, and received a favourable opinion, from The Open University Human Research Ethics Committee (reference number: HREC/3605/Aristeidou), http://www.open.ac.uk/research/ethics/. For the recruitment of a diverse audience, we followed a snowball sampling method, forwarding our invitation to PhD student groups in a number of UK-based universities, but also exploited the reach of PhD social media channels and online PhD groups, and we invited academics and respondents to recruit other participants. Vouchers were provided as an incentive for participation to the first 300 respondents. Before completing the survey, the respondents were provided with an online information sheet and were asked to provide their written consent through a digital consent form. They reported their email addresses to be identifiable and contactable for validation, consent issues, potential withdrawal, and incentive processing. The dataset was anonymized on the 30th of August 2020, prior to initiating data analysis.

Exclusion criteria included survey respondents who ‘straight-lined’ (chose the same answer option repeatedly), gave inconsistent responses to similar questions, or did not use their institution emails (rendering them unidentifiable). Finally, there were 1790 PhD students in the study from 94 different HE institutions across all four UK nations (England, Scotland, Northern Ireland and Wales). The majority of the study population (86%) reported that their research progress had been impacted in a negative way. The dataset [48] included 44.4% male and 55.4% female participants, while the doctoral students in the UK consist of 51% male and 49% female students [17]. Weighting adjustments were made to correct the sample representativeness. The majority of the survey respondents were 25–34 years old (80.4%), with live-in children (71%). Most respondents (86.7%) were conducting their PhDs full-time, and almost two-thirds (64.4%) were funded by a research council or a charitable body in the UK. At the time of the survey, a large proportion of the survey respondents were in the ‘executing’ phase of their research (i.e., data collection/analysis). Finally, a natural science-related PhD was being pursued by slightly over two-thirds of the respondents (68.8%). According to data sourced from HESA [17], the likelihood of individuals embarking on a research postgraduate degree at a younger age (such as 18–20) appears to be relatively low. This is evident from the fact that only 90–130 students within this age group register for such programs each year. More details on the demographics and characteristics of the sample can be found in Table 1 and below.

**Table 1:** Demographics and characteristics of PhD students (N = 1780) in percentages (%).

Demographics			
	%		%
Gender		Live-in partner	
Female	55.4	With	77.9
Male	44.4	Without	21.6
Other	0.2	Prefer not to say	0.3
Age		Live-in children	
18–24	3.3	Yes	71.0
25–34	80.4	No	29.0
35–44	14.0		
45–54	1.8		
55–64	0.5		
**PhD Characteristics**			
PhD mode		Scientific discipline	
Full-time	86.7	Natural sciences	68.8
Part-time	13.1	Applied sciences	11.5
Prefer not to say	0.1	Social sciences	8.0
		Humanities	5.9
PhD phase		Formal sciences	5.6
Planning	3.7		
Executing	80.0	Interest in HE	
Finishing	13.0	High	87.5
Extension/corrections	3.0	Low	11.7
Prefer not to say	0.3	Other	0.6
Funding		Likelihood of HE	
Funded	87.5	High	83.7
Self-funded	11.7	Low	15.7
Other	0.6	Already in academia	0.3
		Not interested	0.3
Impact reason		Impact result	
Childcare & caring responsibilities	76.5	I work fewer hours	71.1
Personal illness	3.7	I work normal hours, but cannot concentrate	12.1
PhD continuation disruption	3.5	I don’t work on my PhD at all	1.6
Mental health	1.0	Prefer not to say	15.2
Prefer not to say	14.3		
		Impact on research progress	
Extension		Yes	86.0
Yes	93.0	No	14.0
No/I don’t know	7.0		

### Variables and instruments

#### Brief COPE Inventory (BCI)

The BCI [49] is a 28-item self-report questionnaire designed to measure effective and ineffective ways to cope with a stressful life event, and it is the abbreviated version of the original 60-item COPE inventory developed by [42]. The BCI has a 4-point Likert scale with options on each item ranging from 0 (I usually do not do this at all) to 3 (I usually do this a lot). Coping in this study is categorised in two overarching coping behaviours, as per Eisenberg et al. [50]: (a) the approach behaviours that attempt to reduce stress by alleviating the problem directly, which include 12 items related to active coping, positive reframing, planning, acceptance, seeking emotional support, and seeking informational support; and (b) the avoidant coping behaviours that attempt to reduce stress by distancing oneself from the problem, which include 12 items related to denial, substance use, venting, behavioural disengagement, self-distraction, and self-blame. Items that belong to neither overarching behaviour are coping related to humour and religion. These were included in the overall coping score but excluded from the analysis based on the two overarching behaviours. A higher score indicates frequent use of that coping behaviour. Cronbach’s alpha for the BCI was .88. Further, both the approach and avoidant scales have shown very good internal consistency in this sample, with Cronbach’s alpha equal to 0.83 and 0.80, respectively.

#### Patient health questionnaire eight-item depression scale (PHQ-8)

PHQ-8 [57] is an eight-item version of the Patient Health Questionnaire (PHQ-9). PHQ is a popular measure for assessing depression and is frequently used for PhD mental health (e.g., [12, 51]), making it an ideal choice for our study. PHQ-9 has been validated as both a diagnostic and severity measure [52, 53] in population-based settings [54] and self-administered modes [55, 56], and it was recently used in a global survey of PhD students’ depression prevalence [12]. PHQ-8 omits the ninth question that assesses suicidal or self-injurious thoughts, and it was deemed more appropriate for our research because researchers in web-based interviews/surveys are unable to provide adequate interventions remotely. The PHQ-8 items employ a 4-point Likert scale with options on each item ranging from 0 (not at all) to 3 (nearly every day). Then, the scores are summed to give a total score between 0 and 24 points, where 0–4 represent no significant depressive symptoms, 5–9 mild depressive symptoms, 10–13 moderate, 15–19 moderately severe, and 20–24 severe [55]. Evidence from a large-scale validation study [57] indicates that a PHQ-8 score ≥ 10 represents clinically significant depression. In this study, Cronbach’s alpha for the PHQ-8 was 0.71, indicating a good internal consistency.

#### Performance satisfaction

Performance satisfaction is an 8-item self-report scale designed to measure the students’ self-perceived progress in their PhD research, their confidence in being able to finish on time, and their satisfaction. The scale was successfully used in a PhD student well-being study at the university of Groningen [24] prior to the Covid-19 pandemic. The performance satisfaction 5-point Likert scale responses range from 1 (completely disagree) to 5 (completely agree). The score for each respondent equals the mean score of the 8-item responses. A reliability analysis was carried out on the performance satisfaction scale. Cronbach’s alpha showed the scale to reach acceptable reliability, α = 0.86.

**Significant life events**Significant Life events is a questionnaire designed to capture whether PhD students had experienced any significant life events in the 12 months prior to the survey. This was successfully used in studying PhD students’ mental health at the university of Groningen [24] prior to the Covid-19 pandemic research disruption. Events include the death of someone close, severe problems in personal relationships, financial problems, severe illness of oneself or someone close, being in the process of buying a house, getting married, expecting a child, none of these events, and prefer not to say. Significant life events were used as an incident control variable in this study.

### Statistical analyses

SPSS (Version 25) was used for statistical analysis. In the first phase, descriptive statistics were used to describe the PHQ-8 Depression and coping behaviours of the sample and the distribution of these three variables among demographics, PhD characteristics, and Covid-19-related circumstances (O1). We used a weighting adjustment for gender to correct the survey representativeness for descriptive analysis; females were given a ‘corrective’ weight of 0.88 and males of 1.15.

In relation to O2, Spearman rank correlations were used to examine the degree of association between all of the 28 coping behaviours and PHQ-8 Depression scores. This finding contributed to our understanding of how individual coping behaviours could relate to lower or higher depressive symptoms.

To assess whether the behaviours significant to our study (i.e., those with a negative or the strongest positive PHQ-8 Depression association) were used more frequently by students of a particular demographic group (O2), we used independent-samples t-test and ANOVA. Before assessing the relationship between our variables, outliers, and groups with a sample size smaller than 15 for each group were removed from the tests (e.g., Gender = other; Funding = partially funded; Likelihood in HE = already employed in academia).

In relation to O3, a binary logistic regression analysis was performed to examine whether Covid-19-related circumstances explain significant depression in PhD students, while controlling for demographics, PhD characteristics, and external incidents. Prior to performing the regression analysis, PHQ-8 Depression score outliers, as well as groups with fewer than 10 events per variable (e.g., gender = other; age = 55–64; Impact reason = mental health), were detected and excluded from the dataset. The dichotomous dependent variable was calculated based on PHQ-8 Depression scores smaller than 10 for non-significant depression, and equal or larger than 10 for significant depression. Associations between Depression in PhD students and the independent variables in our dependency model were estimated using odds ratios (ORs) as produced by the logistic regression procedure in SPSS (Version 25). The ORs were used to explain the strength of the presence or absence of significant depression. Wald tests were used to assess the significance of each predictor. A test of the full model against a constant only model was statistically significant, indicating that the predictors as a set reliably distinguished between PhD students who are having or developing significant depression and those who are not (*Χ*^2^(25)  =  405.258, *p < *.*001*). A Nagelkerke R^2^ of .798 indicated a good to substantial relationship between prediction and grouping (68% of variance explained by the proposed model in completion rates). Table 2 presents response percentages about the categorical variables entered in the model, including the two dependent variables (significant depression and non-significant depression).

**Table 2:** Percentages (%) for categorical data entered in the regression model (n = 1433).

Variable	*n* (%)	Variable	*n* (%)
Depression		Scientific discipline	
Significant	1166 (83%)	Natural sciences	1111 (78%)
Non-significant	267 (17%)	Social sciences	77 (5%)
		Humanities	62 (4%)
Funding extension		Formal sciences	57 (4%)
With	1373 (96%)	Applied sciences	126 (9%)
Without	60 (4%)		
		PhD Phase	
Impact reason		Planning	27 (2%)
Personal illness	55 (4%)	Executing	1247 (87%)
PhD continuation disruption	38 (3%)	Finishing	119 (9%)
Caring responsibilities	1340 (93%)	Extension	28 (2%)
Impact result		PhD mode	
Fewer hours	1239 (86%)	Full-time	1299 (91%)
Normal hours/no concentration	194 (13%)	Part-time	134 (9%)
Not working at all	12 (1%)		
		Funding mode	
Gender		Funded	1308 (91%)
Male	709 (49%)	Self-funded	125 (9%)
Female	724 (51%)		
		Interest in HE	
Age		High	1289 (90%)
18–24	45 (3%)	Low	144 (10%)
25–34	1187 (83%)		
35–44	185 (13%)	Likelihood of HE	
45–54	16 (1%)	High	1354 (94%)
		Low	79 (6%)
Live-in partner			
With	1219 (85%)		
Without	214 (15%)		
Live-in children			
With	1158 (81%)		
Without	275 (19%)		

## Results

### Exploring depression prevalence and coping behaviours

**Fig 1:**
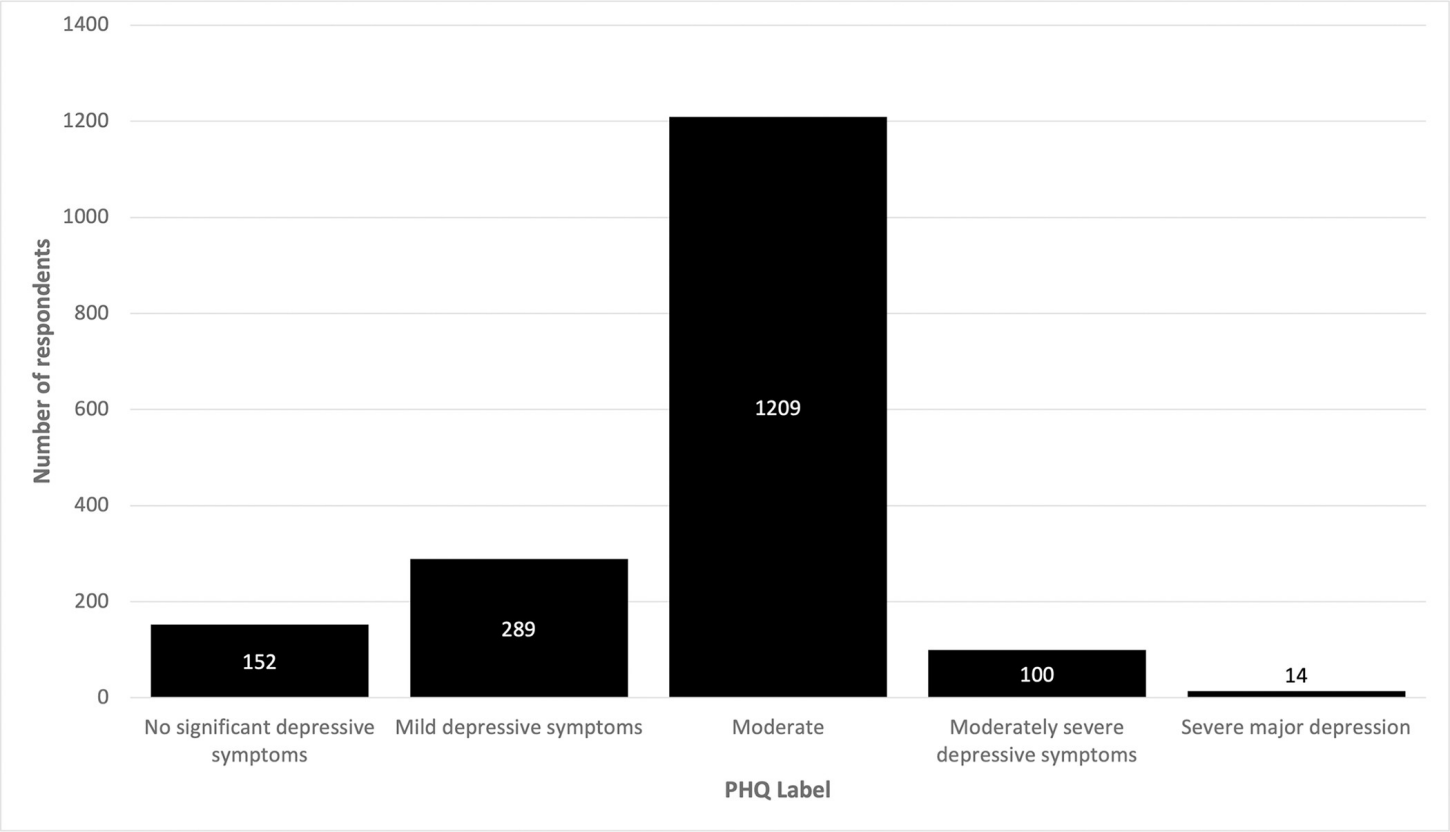
PHQ-8 depression frequency scores.

The average PHQ-8 Depression score was 10.13 (*SD* = 3.23) on a scale of 0–24 (weighted cases). Importantly, this highlights that the majority of survey respondents are facing moderate depression symptoms (Fig 1). The PHQ-8 item with the highest score, in a range of 0–4, was ‘having trouble to concentrate on things, such as reading the newspaper or watching television’ (*M* = 1.45; *SD* = 0.84), and the item with the lowest score was ‘moving or speaking so slowly that other people could have noticed; or the opposite–being so fidgety or restless that have been moving around a lot more than usual’ (*M* = 1.10; *SD* = 0.75). Of the study population, 75% self-reported significant depression (moderate, moderately severe, or severe major).

**Fig 2:**
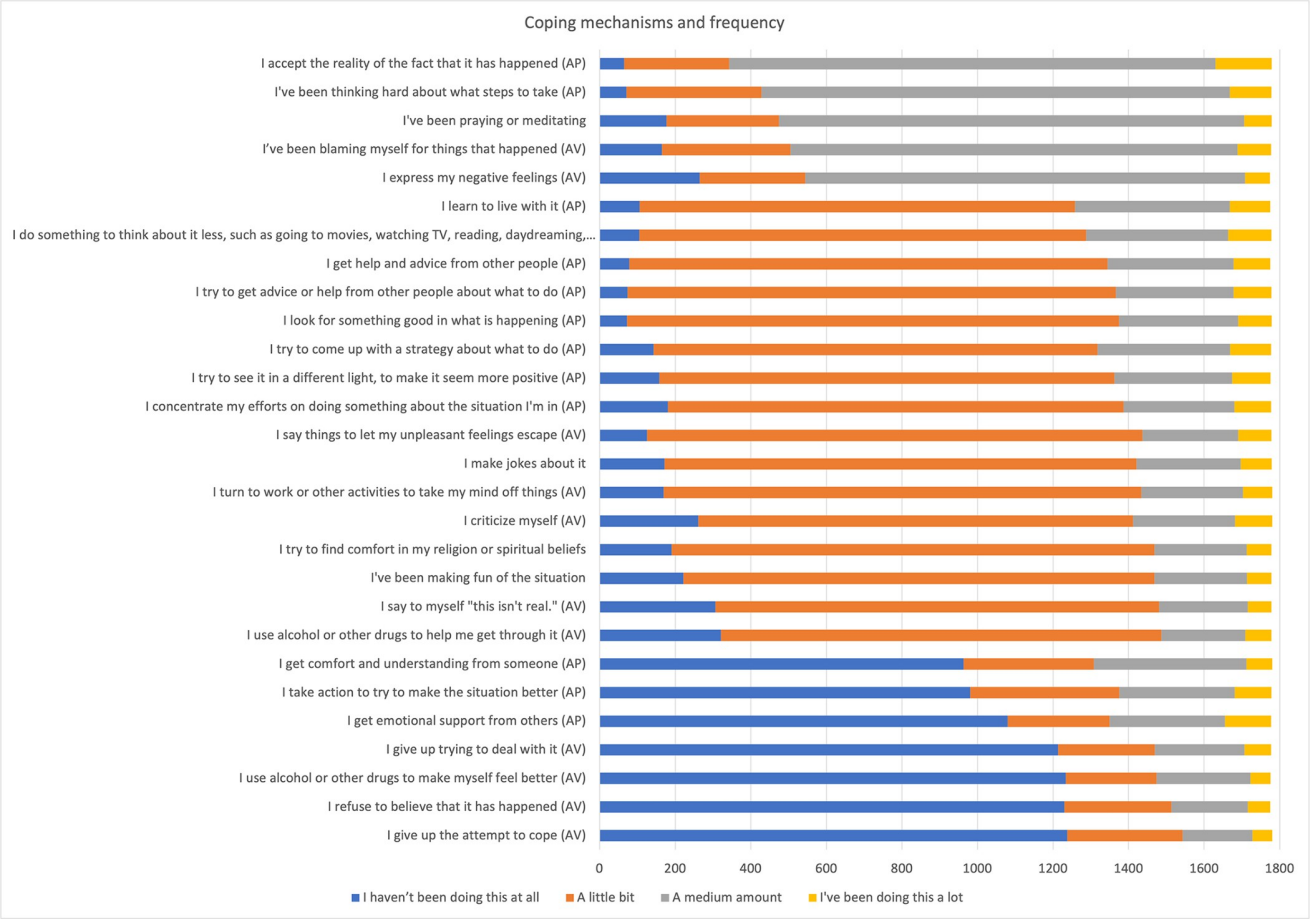
Coping behaviour items and frequency. AV = Avoidant; AP = Approach.

The coping behaviours that the majority of PhD students used in a medium or large amount to overcome the Covid-19 disruption were “accepting the reality of the fact that it has happened” (84%), followed by “thinking hard about what steps to make” *(76%)* (Fig 2). Both are approaching coping behaviours. Other coping behaviours used to a great extent were “praying or meditating” *(73%)*, “blaming myself for things that happened” (avoidant) *(71%)*, and “expressing my negative feelings” (avoidant) (69%). On the other hand, coping behaviours that were used the least were all avoidant ones: “giving up attempting to cope” (*13%)*, “refusing to believe that it has happened” *(15%)*, “using alcohol or other drugs to make myself feel better” *(17%)*, and “giving up trying to deal with it”*(17%)*. Overall, approach coping behaviours were used to a greater extent (*M* = 26.43, *SD* = 5.15) than avoidant coping behaviours (*M* = 23.97, *SD* = 4.90).

The Spearman correlations between coping behaviours and PHQ-8 scores (Table 3), which included outliers, suggested that only two items have significant negative (very weak) associations with depression: Item 15, “getting comfort and understanding from someone” (*r*_s_(1780) = -.107, *p* < .01); and Item 7, “taking action to try to make the situation better” (*r*_s_(1762) = -.077, *p* < .01). The majority of the coping behaviours had a significant positive relationship with higher scores in depressive symptoms. The coping behaviours with the largest effect and a moderate to strong association were Item 13, “criticizing myself” (*r*_s_(1762) = .452, *p* < .01), followed by Item 11 “using alcohol or other drugs to help me go through it” (*r*_s_(1762) = .387, *p* < .01).

**Table 3:** Spearman correlations among coping behaviour items and PHQ-8 depression scores.

Coping behaviour	Spearman correlation PHQ-8
(1) I turn to work or other activities to take my mind off things	0.31**
(2) I concentrate my efforts on doing something about the situation I’m in.	0.16**
(3) I say to myself "this isn’t real."	0.30**
(4) I use alcohol or other drugs to make myself feel better.	0.18**
(5) I get emotional support from others	0.05
(6) I give up trying to deal with it	0.21**
(7) I take action to try to make the situation better.	-0.08**
(8) I refuse to believe that it has happened	0.14**
(9) I say things to let my unpleasant feelings escape	0.17**
(10) I get help and advice from other people	0.12**
(11) I use alcohol or other drugs to help me get through it	0.39**
(12) I try to see it in a different light, to make it seem more positive	0.23**
(13) I criticize myself	0.45**
(14) I try to come up with a strategy about what to do	0.23**
(15) I get comfort and understanding from someone	- 0.11**
(16) I give up the attempt to cope	0.19**
(17) I look for something good in what is happening	0.04
(18) I make jokes about it	0.25**
(19) I do something to think about it less, such as going to movies, watching TV, reading, daydreaming, sleeping, or shopping	0.06*
(20) I accept the reality of the fact that it has happened	0.03*
(21) I express my negative feelings	0.34**
(22) I try to find comfort in my religion or spiritual beliefs	0.17**
(23) I try to get advice or help from other people about what to do	0.06*
(24) I learn to live with it	0.003
(25) I’ve been thinking hard about what steps to take	0.23**
(26) I’ve been blaming myself for things that happened	0.30**
(27) I’ve been praying or meditating	0.18**
(28) I’ve been making fun of the situation	0.33**

Spearman correlations between coping behaviours and PHQ-8 scores. The majority of the coping behaviours had a significant positive relationship with higher scores in depressive symptoms.

*p < .05

**p < .01, two tailed.

Table 4 shows the relationship among approach and avoidant coping behaviours, and demographics. Our analyses indicated that both approach and avoidant coping behaviours had been significantly used to a greater extent by the female over male PhD students, by students without a live-in partner than those with a live-in partner, and by those without live-in children than those with live-in children. There is no evidence that the students of a particular age group were using avoidant coping more than those of another age group. However, students aged 25–34 were using approach coping behaviours less than other groups, and those aged 45–54 more (Table 5).

**Table 4:** Relationship among approach and avoidant coping behaviours, and demographics.

Demographics	Approach Coping M (SD)	t-test/ANOVA	Demographics	Avoidant Coping	t-test/ANOVA
Gender			Gender		
Female (*n* = 977)	27.48 (5.63)	*t* = -10.04, *p* < .001	Female (*n* = 977)	24.31 (5.46)	*t = -*3.33, *p* < .001
Male (*n* = 803)	25.15 (4.16)		Male (*n* = 803)	23.55 (4.09)	
Age			Age		
18–24 (*n* = 57)	28.77 (5.21)		18–24 (*n* = 55)	23.29(4.21)	
25–34 (*n* = 1416)	25.61 (4.44)	F(3,1743) = 45.70, *p* < .001	25–34 (*n* = 1374)	23.49(3.42)	F(3,167) = 2.40
35–44 (*n* = 244)	28.23 (5.28)		35–44 (*n* = 225)	22.76(6.61)	
45–54 (*n* = 30)	32.03 (4.72)		45–54 (*n* = 24)	24.21(6.35)	*p* = .07
Living in partner			Living in partner		
With (*n* = 1371)	25.06 (4.20)	*t* = -20.16, *p* < .001	With (*n* = 1326)	22.55 (3.55)	*t* = -15.86, *p* < .001
Without (*n* = 376)	30.26 (4.49)		Without (*n* = 351)	26.55 (4.36)	
Living in children			Living in children		
With (*n* = 1258)	24.69(3.91)	*t* = -22.79, *p* < .001	With (*n* = 1215)	22.43 (3.32)	*t* = -14.35 *p* < .001
Without (*n* = 498)	30.07(4.66)		Without (*n* = 471)	25.86 (4.75)	

The relationship among approach and avoidant coping behaviours, and demographics indicated that both approach and avoidant coping behaviours have been significantly used to a greater extent by specific student groups.

**Table 5:** ANOVA post-hoc comparisons of approach coping scores between age groups.

	Approach coping			Games-Howell Comparisons		
Group	n	Mean	SD	18–24	25–34	35–44
18–24	57	28.77	5.21			
25–34	1416	25.61	4.44	*p* < .001		
35–44	244	28.23	5.28	*p* = 0.89	*p* < .001	
45–54	30	32.03	4.72	*p* = 0.02	*p* < .001	*p* < .001

The table shows the results of the ANOVA post-hoc comparisons between age groups. Students aged 25–34 were using approach coping behaviours less than other groups, and those aged 45–54 more.

Our analyses indicated that female PhD students, who had significantly lower PHQ-8 Depression scores, were using Table 3‘s Items 15 (*t*[1778] = 14.61, *p* < .001) and Item 7 (*t*[480] = 15.11, *p* < .001) significantly more than male students. Also, those without live-in partners were getting comfort and understanding from someone to a significantly greater extent than those without (*t*[702] = 20.09, *p* < .001). PhD students without live-in children were taking action to try to make the situation better significantly more than those who have them (*t*[894] = 25.21, *p* < .001).

### Predictors of depression and relative influence

Covid-19-related circumstances (receiving an extension, impact reasons, and impact results), performance satisfaction, and coping behaviours (approach and avoidant) were entered together as predictors of depression. Demographics (gender, age, live-in partner, and live-in children), PhD characteristics (discipline, PhD phase, PhD mode, funding, interest in HE, and likelihood in HE) and external incidents were used as control variables. Table 6 reports the findings of the analyses.

**Table 6:** Predictors of depression in PhD students during the Covid-19 pandemic, UK 2020 (N = 1433).

Predictor	B	S.E. (B)	Wald	*p* value	OR
Constant	-8.03	2.86	7.89	.005**	< .001
**Covid-19 circumstances**					
Without extension	1.69	0.69	6.03	.014*	5.41
Impact reason					
Personal illness (ref)					1.00
PhD continuation disruption	0.42	1.05	0.155	0.69	1.51
Caring responsibilities	-2.31	0.66	12.50	< .001***	0.10
Impact result					
Fewer hours (ref)					1.00
Normal hours but cannot concentrate	-0.07	0.31	0.05	0.83	0.94
Not working at all	-1.90	1.45	1.72	0.19	.15
Performance satisfaction	-0.42	0.22	3.55	0.06	0.66
Approach coping behaviours	-2.01	0.54	14.03	< .001***	0.13
Avoidant coping behaviours	3.78	0.56	45.81	< .001***	43.73
**Incident control variable**	1.81	0.19	92.50	< .001***	6.13
**Demographics**					
Male	0.66	0.29	5.26	.047*	1.34
Age					
18–24 (ref)					1.00
25–34	-2.97	1.80	2.72	0.10	0.51
35–44	-1.56	1.67	0.87	0.35	0.21
45–54	-4.56	1.70	7.22	0.07	0.01
Live-in partner	1.92	0.50	14.54	< .001***	6.80
Live-in children	2.63	0.51	26.26	< .001***	13.88
**PhD characteristics**					
Scientific discipline					
Natural sciences (ref)					1.00
Social science	-2.27	0.50	21.02	< .001***	9.68
Humanities	0.41	0.66	0.38	0.54	1.50
Formal sciences	1.63	0.67	5.91	0.015*	5.12
Applied sciences	0.78	0.71	1.21	0.27	2.19
PhD phase					
Planning (ref)					1.00
Executing	1.68	0.85	3.91	0.048*	5.34
Finishing	1.20	1.13	1.13	0.29	3.33
Extension	0.43	0.90	0.23	0.63	1.54
PhD mode: Full-time	-0.48	0.50	0.92	0.34	0.62
Funded	1.01	0.45	4.99	0.052	2.75
High interest in HE	1.20	0.48	6.29	0.012*	3.35
High likelihood of HE	1.32	0.61	4.62	0.03*	3.73

Note. ref = reference category

* = p < 0.05 ** = p < 0.01 *** = p < 0.001, Model fit PHQ-8 LR = 405.258 df = 26 p < 0.001 Nagelkerke R^2^ = 0.798

Prediction success overall was 95.3% (83.1% for not significant depression and 98.0% for significant depression). The Wald criterion demonstrated that not having an extension (*p* = .014), having caring responsibilities (*p* < .001), and using approach (*p* < .001) or avoidant (*p* < .001) coping behaviours made significant contributions to prediction. The OR value indicated that in the case that PhD students were not receiving an extension amid the Covid-19 disruption, or they did not know whether they were receiving one yet, they were 5.4 times more likely to experience significant depression. For the impact reason, our findings showed that–compared to those who experienced personal illness–PhD students who had caring responsibilities (e.g., childcare or other) showed slightly lower depressive symptoms (OR = 0.10). The OR for approach and avoidant coping behaviours were 0.13 and 43.73, respectively. This finding indicates that when approach coping is raised by one unit (e.g., +1 to the score), we see evidence for better mental health, while when avoidant coping is raised by one unit, a PhD student is very likely (44 times) to experience significant depression.

Turning to our control variables, PhD students with children in the household and with live-in partners showed significantly higher odds (about 14 and 7 times more, respectively) of having or developing depressive symptoms than those without. The latter can be explained by the fact that 88% of the participants with live-in partners also reported having live-in children. Also, male students were slightly more likely than female students to experience significant depression (with a borderline p-value), but this might be explained by the significantly increased use of coping approaches by female students. This gender-related finding that shows nearly no difference between the two categories slightly differs from Goldstone and Zhang’s model [15] which highlights a difference between female and male participants’ mental wellbeing. This difference can be explained by the fact that the research instruments used in the two studies were different, as well as the survey period.

Some PhD characteristics that made significant contributions to prediction were the discipline of PhD studies and the interest of students to remain in academia after finishing their PhD projects. The risk of experiencing significant depression in PhD students in social sciences (OR = 9.68) was lower than in students conducting a PhD in natural sciences. In contrast to findings by Levecque et al. [9], we observed that PhD students expressing a high interest in an academic career were 3.5 times more likely to develop depressive symptoms than those with no or only little interest in remaining in academia. Further, those considering having a high likelihood of remaining in academia were slightly more depressed (OR = 3.73), as well as those who were in the executing phase of their PhD research (OR = 3.33). No differences between funded and self-funded students were detected. Finally, the OR for the external incident variable was 6.13, indicating that for each incident unit (e.g., one more incident), we see evidence for depressive symptoms that are six times worse.

## Discussion

Our study contributes new empirical data and new insights needed to develop knowledge on the effect of university research disruption on the PhD student population. In turn, new knowledge may provide the evidence base for university and research policy.

### Exploring mental health and coping behaviours

Our first contribution is to provide empirical estimates for the performance satisfaction, prevalence of mental health problems, and coping behaviours of PhD students during the pandemic-induced research disruption, on the basis of representative data across disciplines and across universities in the UK.

Our findings show that most UK PhD students across universities and disciplines report that their research progress has been affected negatively (86%). By contrast, in pre-pandemic periods, 79% of UK PhD students across Universities and disciplines had indicated excellent research progress [11]. This shift within the same population is important to reveal because of its potential implications for PhDs’ careers and university research capacity and innovation, as we know that dissatisfaction about the PhD trajectory is tied to negative outcomes such as attrition and delay [24, 28], but also to lower productivity [58].

We found that during the period of severe research disruption caused by the Covid-19 pandemic, 75% of the UK students surveyed from 94 universities and across disciplines self-reported in the moderate-severe range for depression. This is at least three times more compared to the reported prevalence of depression among the general population internationally during the Covid-19 outbreak (16–28%, [59]). Our findings are also in line with findings in Goldstone and Zhang’s study [15] on UK postgraduate students’ mental wellbeing during the pandemic, in which 72% of the surveyed students were found to demonstrate possible or probable depression or anxiety.

By adopting widely used standardised questionnaires, our findings provide an accessible benchmark for the comparison with studies that took place among PhD student populations in periods of HE stability (pre-2020), thereby providing the empirical basis to accurately estimate the issue of poor mental health among PhD students during a period of research disruption. Using the same questionnaire as in our survey (PHQ-9) and drawing on a sample of PhD students from multiple universities and across research disciplines, a pre-pandemic global survey reported that 39% of PhD students scored in the moderate-severe range for depression [12]. Pre-pandemic national surveys of PhD students across institutions and disciplines report similar rates of depression, between 32% (in Belgium, Levecque et al. [9] and 38% (in the Netherlands, Van der Weijden et al. [60]. In a pre-pandemic (2018–2019) survey of UK PhD students across 48 universities and disciplines, only 25% reported levels that would indicate probable depression or anxiety [11]. These comparisons indicate that the prevalence of depression among the UK PhD student population of our study during the pandemic-induced period of research disruption is two-to-three times more than that which was reported in periods of stability for the UK PhD student population, for PhD student populations of other countries, and the global PhD population.

Our findings on PhD students’ mental health and PhD students’ coping advance past literature [22, 23, 34] in two significant ways. First, by using a highly reliable coping measure (COPE), we are able to demonstrate the relationship between coping styles and mental health outcomes in PhD students in a way that allows for comparisons and to build further research in this area. Second, we identify specific coping behaviours amongst the UK PhD students that are associated with lower depression scores and some that have a negative association with depression (i.e., *getting comfort and understanding from someone* and *taking action to try to make the situation better*). Both are ‘coping approach’ behaviours (i.e., attempts to reduce stress by alleviating the problem directly; [50]). Studies using COPE in other populations have also linked coping-approach behaviours to fewer symptoms of psychological distress [45], more physical and psychological well-being at work [46], and an absence of anxiety and depression [61].

## Factors explaining PhD students’ depression

Our second contribution is to explain–within the UK PhD population–whose mental health is more affected by the pandemic-induced research disruption. We find that several factors have a significant impact on PhD students to have or develop mental health issues during a period of research disruption.

Consistent with past research on PhD students’ mental health, our findings reveal the significant influence of their personal lives on poor mental health. The relationships we observed during a period of research disruption, however, differ from those suggested in studies conducted in periods of stability (e.g., [9, 22, 25, 26, 62]). We found that PhD students with live-in children or with a live-in partner and PhDs with caring responsibilities are more likely to have or develop significant depression compared to those without. This difference can be explained by the closure of schools that resulted in parents home-schooling their children, a greater demand for devices and the internet in households, and parents going through emotional hardship [63]. We additionally find six times worse depressive symptoms for each ‘external life incident’ (e.g., childbirth, moving home) that occurred in the PhD students’ lives. A larger number of external incidents were found to be associated with students with live-in partners and students with live-in children, which may explain these as reinforcing negative effects. These new insights explain that–although most of these realities in PhD students’ personal lives existed besides the research disruption—when combined with the research disruption, their mental health can spiral downward.

Our findings also address the role of structural PhD characteristics (PhD discipline and PhD phase) in predicting whether a student might present mental health issues in times of research disruption. We find that in a period of research disruption, the risk of significant depression is higher in the execution phase of the PhD compared to the beginning or extension phases, contrary to Levecque and colleagues’ findings [9]. Because there is very limited research on the PhD stage and mental health, our findings contribute insights to a broader community of scholars who advocate for the further study of the challenges in each PhD stage discreetly (e.g., [32]). Furthermore, we find that the risk of experiencing significant depression in PhD students in social sciences was lower than students conducting a PhD in natural sciences. Our survey respondents offered explanations on the role of PhD discipline in mental health during the pandemic in the open text responses. These converge on the fact that natural sciences often require being physically in a laboratory, which is probably unfeasible when university facilities are closed.

In tune with past research on finances and mental health in PhD students [9, 64], we found those without funded extensions are more likely to have or develop significant depression (moderate, moderately severe, and severe) compared to those with them. We reveal the size of this association (about 5.5 times more) and link PhD funding extensions to standardized assessments of depression prevalence, thus uniquely providing new evidence for policy scholars.

### Implications for research and higher education policy

Our findings show an alarming increase in self-reported depression levels among the UK PhD student population. The long-term mental health impact of Covid-19 may take years to become fully apparent, and managing this impact requires concerted effort not just from the healthcare system at large [59] but also from the HE sector specifically. With mental illness a cause for PhD student attrition, loss of research capacity and productivity, data from our survey should prompt consideration of immediate intervention strategies.

For research and education policy scholars, our findings contribute directly to the development of evidence-based research and university policies on support for targeted groups of PhD students in times of disruption. Specifically, our findings show that institutional and funder support should not only be in the form of PhD-funded extensions–which are nevertheless shown in our study and other studies (e.g., [15]) to be very significant. But also, in the form of providing expedited alternatives to the changes evoked by the pandemic for PhD students, such as new and adjusted policies that explicitly consider those PhDs with caring responsibilities, since 77% of our respondents reported that childcare and other caring responsibilities are the reason for dissatisfaction with their PhD progress. If not, the Covid-19 research disruption could erase decades of progress towards equality in academia [65].

Our main contribution is that we offer insights into how to mitigate mental health consequences for PhD students in times of research disruption. Individual-driven coping behaviours are suggested to be of equal importance to those promoted by the PhD students’ institutions [66]. In this study, approach coping behaviours were found to associate with lower depression levels, which may eventually contribute to PhD completion. The importance of developing coping mechanisms has also been highlighted in pre-pandemic studies, with, for instance, mothers finding ways to combine academic work and family responsibilities and succeed in both roles [38]. Still, institutions may play a crucial role in offering training for PhD students on coping and wellbeing through, for instance, a virtual platform to comply with social distancing policies. Such efforts may include mental health support and coping behaviour guidance, so that students are guided on how to successfully deal with disruptions (for example, to avoid avoidant coping behaviours that may lead them to higher levels of depression). Pre-pandemic reforms have previously shown that a well-structured programme and well-timed financial support can facilitate and uphold PhD completion, alongside student efforts [35]. As the future generation of academics, PhD students would be better equipped to handle the current and future disruptions and better cope with other disruptions in their academic journeys.

### Limitations and implications for further research

Although our study has gone some way towards enhancing our understanding of Covid-19-related effects on UK PhD students’ mental health, it is plausible that a number of limitations could have influenced the results obtained. First, while our research attracted a representative number of students from different age groups, PhD modes, phases and funding, there was a very strong presence of students in natural sciences [17]. Second, as this was a cross-sectional study, we did not follow the UK PhD population longitudinally, and we may not offer insights into the trajectory of the relationships we articulate in our findings. Nevertheless, our adoption of standardized questionnaires allows for a platform for comparisons with past and future research efforts. Third, findings in this survey are based on self-report and may be subject to unconscious biases (e.g., PhD students assessing themselves or the situation inaccurately). Fifth, the research undertaken employed the PHQ-8 with a specific emphasis on assessing aspects related to depression. It is important to acknowledge that while these questionnaires offer valuable insights into depression, they may not comprehensively encompass the broader spectrum of general mental health. Therefore, the findings of the study should be interpreted within the context of its targeted focus on depression, recognizing the potential existence of other dimensions of mental health that were not directly addressed within this research framework. Finally, despite the high percentage of prediction in our findings (80%), additional factors may likely explain variabilities in our study outcomes, such as leadership factors or supervision styles in the 94 UK Universities whose PhD students participated in our survey.

As our study strongly demonstrates, juxtaposing findings from studies conducted during periods of relative HE stability with those conducted during periods of disruption is a fruitful approach for advancing research and university policy. By identifying which insights that would have been invaluable during periods of stability are less so during a period of disruption, scholars can provide significant advancements to existing research and new insights for policy, research and HE leadership.

## Conclusions

Our study extends previous research on mental health in the HE sector by considering the dynamics of a severe disruption as opposed to the dynamics of relative stability in PhD mental health and coping behaviours. Drawing on our insights into these interrelationships, we suggest extensions to the literature on PhD students’ mental health, research and university policy. With our findings, HE leaders and policymakers may be better placed to tackle and ultimately overcome this and future research disruptions.
