# Correction: Research disruption during PhD studies and its impact on mental health: Implications for research and university policy

**DOI:** 10.1371/journal.pone.0311017

**Published:** 2024-09-19

**Authors:** The PLOS ONE Editors

## Notice of Republication

This article [1] was republished on August 19, 2024, to temporarily update the Data Availability statement while editorial follow-up regarding the article’s data availability was ongoing. The temporary Data Availability statement was: PLOS is discussing with the authors issues pertaining to data availability; in the meantime, requests about underlying data should be sent to the corresponding author.

The article was republished again on September 12, 2024, to restore the original Data Availability statement after editorial follow-up concluded. Please download this article again to view the correct version.
